# Contributions of Indoor and Outdoor Sources to Ozone in Residential Buildings in Nanjing

**DOI:** 10.3390/ijerph16142587

**Published:** 2019-07-19

**Authors:** Yu Huang, Zhe Yang, Zhi Gao

**Affiliations:** School of Architecture and Urban Planning, Nanjing University, 22 Hankou Road, Nanjing 210093, China

**Keywords:** ozone, contributions, CONTAM, I/O ratio, respiratory exposure

## Abstract

Ozone has become one of the most serious air pollutants in China in recent years. Since people spend most of their time indoors, the ozone in the indoor environment could be a major factor affecting the occupants’ health. The indoor ozone in residential buildings mainly comes from two sources: outdoor atmosphere and indoor ozone produced by electrical devices. In this study, a typical residence in Nanjing was taken as an example to calculate and compare the contributions of indoor and outdoor sources to ozone in the building. A questionnaire survey about the type, the placement, and the frequency of use of the ozone emission devices was performed to provide the basis for the settings of indoor ozone sources. The multi-zone software CONTAM was used hourly to simulate the ozone concentration in summer and in winter with inner doors either closed or open, and it was noted whether there were ozone emission devices indoors or not. Source contribution was quantified and compared by three methods in this paper: (1) the average indoor/outdoor (I/O) ratio, (2) the I/O ratio frequency, and (3) the ratio of indoor ozone concentration without ozone sources to that with ozone sources. The results showed that the contribution of outdoor sources was much greater than that of indoor sources in summer, but in winter, the frequency of I/O > 1 could reach 55.8% of the total seasonal time, and the ratio of indoor ozone concentration without sources to that with sources could reach as high as 74.3%. This meant that the indoor concentration had the potential to exceed the outdoor. Furthermore, human respiratory exposure in different ages and genders was calculated. It was found that teenagers aged 10–18 years old and female adults had a higher respiratory exposure level.

## 1. Introduction

In recent years, with the rapid development of economy and the acceleration of urbanization, air pollution in China has continued to increase. This not only influences people’s health outdoors but also affects the indoor environment through windows and doors. Ozone has gradually become one of the most serious air pollutants in China, and it may cause severe photochemical pollution. The average ozone concentration in Nanjing has increased by over 25% in the last five years [1]. Numerous studies have suggested that too much exposure to ozone may cause respiratory morbidity, impaired lung function, local or systemic inflammation, cardiovascular disease, and cancer in human beings [2,3,4,5,6,7].

There are two main sources of ozone in residential buildings—the outdoor sources that enter the room mainly through natural ventilation, mechanical ventilation, and infiltration through cracks in the building envelope [8], and the indoor sources that are emitted by some electrical devices. Ground-level ozone is mainly produced by photochemical reactions of oxygen, nitrogen oxides (NOx), and volatile organic compounds (VOCs) in the atmosphere. The commonly used indoor ozone emission devices in the literature include photocopying, air purifying, disinfecting devices, etc. [9,10,11,12,13,14,15]. The ozone emission mechanisms can generally be classified into two categories: photochemical mechanisms and corona discharge mechanisms. Guo et al. [14], in reviewing the types of common indoor ozone emission devices and their ozone emission rates, found the mean ozone emission rates of in-duct air cleaners, ozone generators, room air purifiers, photocopiers, laser printers, and other small devices to be 62.8, 76.3, 4.6, 3.3, 0.8, and 0.4 mg/h, respectively. Shen et al. [16] made a literature review on ozone removal from the surface of building materials. They concluded that the reaction probability of common building materials ranged from 10^−8^ to 10^−4^, and the surface-treated materials may be more decisive than the underlying materials on ozone deposition. Various scholars have studied the relationship between indoor and outdoor ozone concentrations. Weschler [17], for example, monitored indoor and outdoor ozone concentration in three office buildings for five months (May–October). Their results showed that indoor ozone concentration fluctuated between 20% and 80% of the outdoor ozone concentration depending on the air exchange rate. Ozone indoor/outdoor (I/O) ratios in other offices can be predicted by air exchange rate using a single-area model. Weschler et al. [18] found that outdoor ozone concentrations varied greatly with places, time, and location. The average outdoor ozone concentration in a day tended to be low in the morning and at night and high at noon. During the year, it tended to be high in spring and summer and low in autumn and winter. The half-life was usually between 7 and 10 min for ozone indoors, which has a relationship with surface removal rate and air exchange rate. Fadeyi [19] proposed that the contribution of ozone air purification equipment with a high ozone emission rate to indoor concentration could easily exceed that of outdoor ozone.

The multi-zone model can solve the problems of air exchange and pollutant transfer between rooms as well as between indoor and outdoor environments [20]. This model assumes that indoor pollutants are uniformly mixed and requires relatively small computing power [21]. Ng et al. [22] in 2012 used the multi-zone program CONTAM to create commercial reference buildings to support airflow and indoor air quality (IAQ) analyses. Ng et al. [23] in 2015 used CONTAM to simulate a big box retail store in two climates to investigate the IAQ and the energy trade-offs of various ventilation approaches. Dols et al. [24] described how CONTAM coupled with EnergyPlus identified the interdependence between airflow and heat transfer and allowed for sharing of data. It can be seen that the multi-zone model represented by CONTAM is very suitable for macro-analysis of the interaction between building zones and has a high degree of agreement with the research object and the content of this paper.

In the comparative study of indoor and outdoor ozone sources contribution, the most important task was to quantify the contribution. Shi [25] developed a theoretical model to analyze contributions of indoor and outdoor sources to indoor airborne polycyclic aromatic hydrocarbons. Wang et al. [26] investigated the spatiotemporal variation and the sources contribution of the air pollutants in three provincial capitals in northern China. Multiple linear regression was used to estimate source contributions using pollutant concentration and the absolute score factors. Ji et al. [27] studied the contribution of different sources of particulate matter to indoor PM_2.5_ concentration in residential buildings. A mass balance model was employed to estimate the different sources’ contributions to the indoor PM_2.5_ concentrations.

This study was focused on the indoor and the outdoor source contributions on the basis of the multi-zone model CONTAM. The ozone concentration of each room would be simulated when there are both sources and only outdoor sources indoors. Indoor ozone sources emit more ozone in a short time but generally do not work continuously for long periods of time. The purpose of this study was to compare the indoor and the outdoor ozone contribution in order to warn people to take appropriate measurements to reduce human ozone exposure.

## 2. Methodology

### 2.1. Physical Model and Parameter Settings

**Figure 1:**
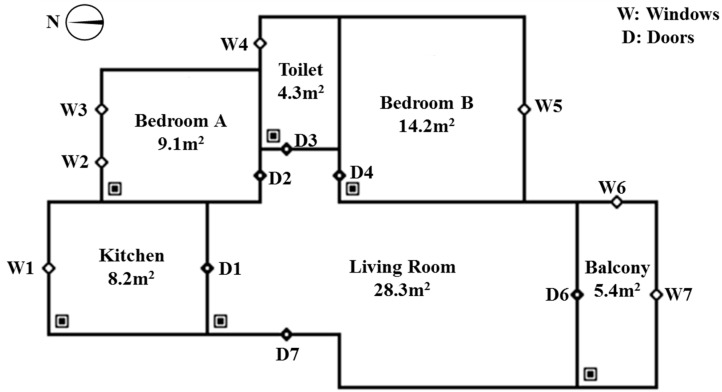
Building model created in CONTAM.

Nanjing (N 31°14’–32°37’, E 118°22’–119°14’), a major city in eastern China, has a population of more than 8 million. According to the statistical yearbook by the government [28], new residential buildings in Nanjing in the past eight years were mainly apartment houses. Among these, apartments with a building area below 90 m^2^ accounted for 58.3%, those between 90 m^2^ and 144 m^2^ accounted for 31.1%, and those over 144 m^2^ accounted for 7.7%. The sixth national census of Nanjing showed that the number of three-person households accounted for the largest proportion, which was 35.83% [29]. Based on the above two points, we took three-person households (i.e., two-bedroom households) as an example and combined the building area range and the Nanjing housing market to determine a typical household type. It is a six-room residence in Nanjing, whose functions, areas, and relative positions of each room are shown in Figure 1. The selection of the building model could only represent part of the housing situation in Nanjing, but the research methods and the results are applicable to most scenarios.

In this paper, the multi-zone simulation software CONTAM (CONTAM 3.2, National Institute of Standards and Technology (NIST), Gaithersburg, MD, USA) was used to create a model of the apartment. The small diamonds on the walls of the building were the openings, including the inner and the outer doors and windows. Their sizes are included in Figure 1. The floor height of residential buildings in Nanjing is generally 2.8–3 m. Except for the girder height and the ceiling height, the net height of this building model is 2.4 m.

This study focused on the two most unfavorable situations for indoor ozone pollution, namely, opening all outer windows in summer and closing all outer windows in winter. Indoor temperatures in summer and winter were set at 26 and 18 °C, respectively, according to air conditioning design standards. Outdoor meteorological parameters in this study were the actual data from meteorological station in Nanjing in 2018. The main wind direction in summer in Nanjing is from the southeast and in winter from the northeast.

The air flow path in CONTAM software was the passageway between zones as well as between zones and atmosphere. Each zone connected to the exterior through windows and doors, while the interior connected only through doors, and the air of each room was well mixed. In this study, we assumed that the air flow in all openings was one-way flow. When doors and windows were opened, the effective ventilation area was equal to the actual opening area. When doors and windows were closed, the small area leakage model was adopted, and the effective leakage area could be calculated according to Equation (1) [30,31].

(1) $$L=\frac{Q_{r}\sqrt{\frac{\rho }{2\Delta P_{r}}}}{C_{d}}$$

where L is effective leakage area (m^2^), $Q_{r}$ is leakage air volume (m^3^/s), ρ is air density (m^3^/kg), $\Delta P_{r}$ is pressure difference (Pa), $C_{d}$ is dimensionless flow coefficient, $\Delta P_{r}$, ρ and $C_{d}$ are usually constant and equal to 10 Pa, 1.27 m^3^/kg, and 0.6, respectively, $Q_{r}$ can be calculated by area index method, and 50% surplus is set aside. The size and the effective area of each opening are shown in Table 1.

### 2.2. Questionnaire Survey on Indoor Ozone Emission Devices

To determine the use of indoor ozone emission devices in residential buildings in Nanjing, an online questionnaire was distributed primarily to long-time Nanjing residents. A total of 316 available questionnaires were collected, each dealing with the use of ozone emission devices. The questionnaire is shown in Table 2. Questions No. 1 and No. 2 are related to the type of the ozone emission devices and their ozone emission rate. No. 3 is about the placement, and No. 4 and No. 5 are designed to determine the frequency of use. Question Nos. 1, 4, and 6 are single option questions, while Nos. 2, 3, and 5 are multiple option questions. Question No. 2 requires filling the blank of device model number.

### 2.3. Contributions Quantification

In this paper, three methods were used to quantify the contribution of indoor and outdoor ozone sources.

I/O was the ratio of indoor ozone concentration to outdoor concentration. It could be expressed by the Equation (2).

(2) $$\frac{I}{O}=\frac{c_{in}}{c_{out}}$$

where $c_{in}$ and $c_{out}$ are indoor and outdoor ozone contribution. When I/O was greater than, less than, or equal to 1, it indicated that the contribution of indoor ozone sources was greater than, less than, or equal to the contribution of outdoor ozone sources.

I/O frequency analysis was based on the I/O ratio. Hourly I/O values in summer and winter were calculated, and the frequency of different I/O intervals was counted. The method of judging contributions was similar to the I/O method. The higher the frequency of I/O > 1, the greater the contribution of indoor sources was.

*P_o_* was the ratio of indoor ozone concentration without indoor ozone sources to that with indoor ozone sources. It could be expressed by the Equation (3).

(3) $$P_{o}=\frac{CONTRI_{\mathrm{out}}}{CONTRI_{\mathrm{in}}+CONTRI_{\mathrm{out}}}=\frac{c_{in,\mathrm{without}\;sources}}{c_{in,with\;sources}}$$

where $CONTRI_{\mathrm{in}}$ and $CONTRI_{\mathrm{out}}$ are indoor and outdoor source contributions, respectively. When there were no ozone sources indoors, it meant that the indoor ozone came entirely from outdoors. Therefore, indoor ozone concentration without indoor ozone sources represented the outdoor source contributions. When there were ozone sources indoors, the indoor ozone was from the combination of indoor and outdoor sources. Thus, $P_{o}$ represents the proportion of outdoor ozone contribution in the total contribution.

## 3. Results and Discussions

### 3.1. Questionnaire Survey on Indoor Ozone Emission Devices

The questionnaire survey showed that 68.4% of the residential buildings had ozone emission devices. Among them, 28.2% of the residences had photocopying devices, 15.4% had disinfecting devices, and 30.8% had air purifying devices, whereas some other ozone emission devices, such as refrigerators with a purification function, shoe cleaners, etc., accounted for a total of 25.6%. This paper mainly focused on the first three kinds of devices for their detailed study.

**Figure 2:**
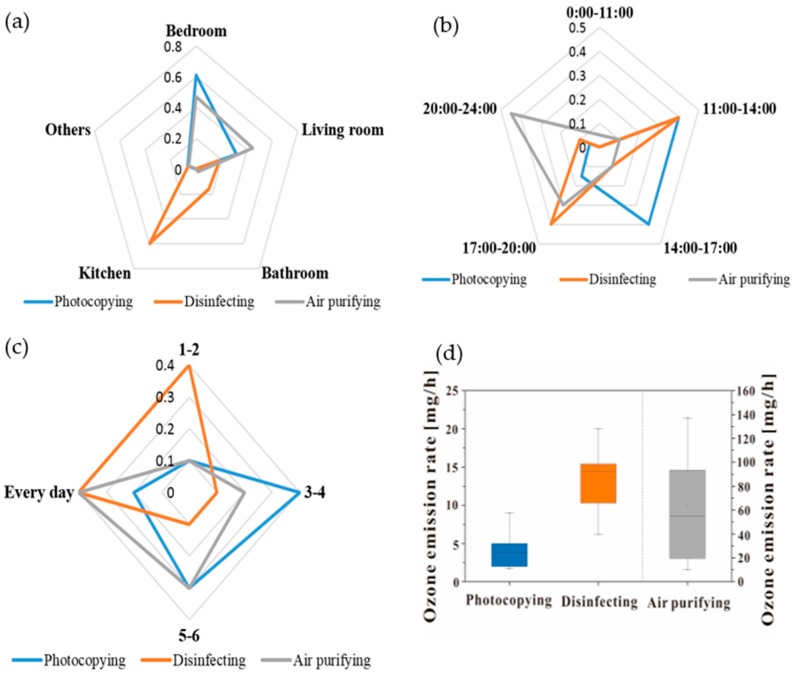
Questionnaire analysis. (**a**) The frequency of the devices in different rooms; (**b**) time period of use in a day; (**c**) frequency of use in a week; (**d**) ozone emission rate.

Figure 2 shows the questionnaire analysis. It can be seen from Figure 2a that photocopying and air purifying devices were mainly located in the bedroom and the living room, and disinfecting devices were mainly placed in the kitchen. This was determined by the function of the devices themselves. Figure 2b shows that most residents used photocopying devices 3–4 times a week and used air purifying devices every day. For disinfecting devices, the percentage of using 1–2 times a week and every day was the same, both accounting for 40%. Figure 2c shows that photocopying devices were often used from 11:00–14:00 and from 14:00–17:00, disinfecting devices were often used from 11:00–14:00 and from 17:00–20:00, and air purifying devices were often used from 20:00–24:00. This was related to residents’ living habits, including diet, sleep, and so on. By obtaining the model numbers of the devices and looking up the information of the products at the factory, ozone emission rates of various kinds of devices were analyzed, as shown in Figure 2d. The ozone emission rates of photocopying devices were 1.7–9.0 mg/h, the rates of disinfecting devices were 6.2–20.0 mg/h, and the rates of air purifying devices were 10–137 mg/h. It was obvious that air purifying devices were the main indoor ozone sources, especially those with an ozone purification function. The results of the questionnaire survey provided basis for the settings of indoor ozone sources.

### 3.2. Natural Ventilation without Indoor Ozone Sources

**Figure 3:**
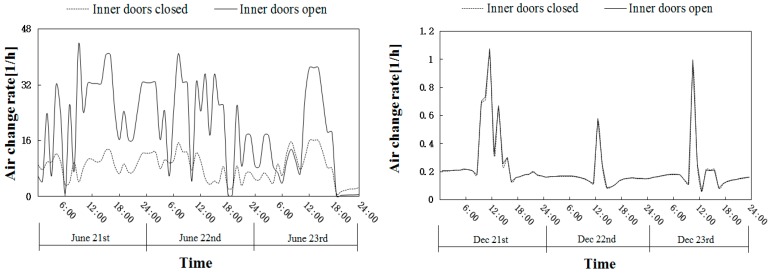
Hourly average air exchange rate of the building in summer and in winter.

The natural ventilation under the two most unfavorable conditions with inner doors both closed and open was studied in detail. Both wind pressure and thermal pressure were considered. The summer condition was represented by three days near the summer solstice (21–23 June), and winter was represented by three days near the winter solstice (21–23 December). The hourly air exchange rate of the building average is shown in Figure 3. It can be seen that the ventilation of the building in summer was much better than that in winter, and the air exchange rate was one order of magnitude larger. It was related to the settings of the two most unfavorable conditions. The air exchange rate in summer had multiple peaks in a day, while in winter, there was usually only one peak in a day, and it occurred around 12:00. The opening of the inner doors in summer could improve the ventilation, but in winter, the ventilation had little to do with the inner doors.

Figure 3 uses the building average as a representative to show the hourly variation of the air exchange rate when there were no sources indoors. The average air exchange rate and the ozone concentration in each room are shown in Table 3. It was found that Bedroom A had the best ventilation; the air exchange rate in summer with inner doors opening could reach as high as 78.58 h^−1^. Ozone concentration also varied with the season and the state of the inner doors. When the inner doors were open in summer, the ozone concentration of all rooms was higher than that when the inner doors were closed, except Bedroom A, since Bedroom A was on the windward side of the building. When the inner doors were open in winter, the concentration of each room increased slightly.

When there were no indoor sources, the indoor ozone came entirely from the outdoors. The natural ventilation pattern without indoor ozone sources served as the basis for calculating the contributions of the sources in the later section.

**Figure 4:**
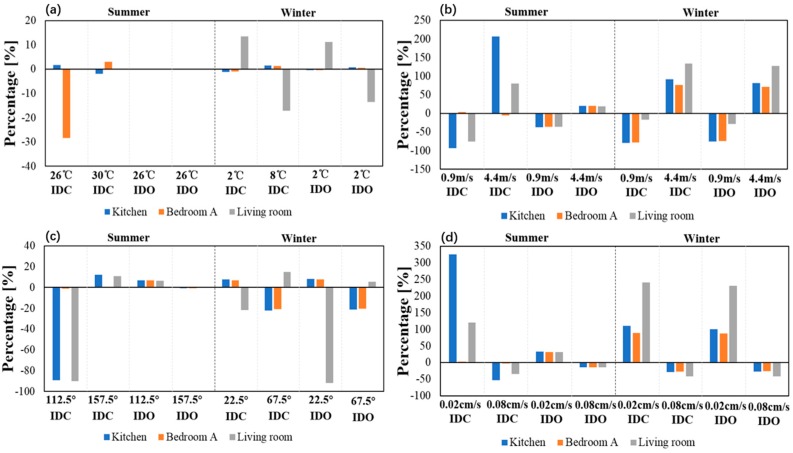
Influencing factors of indoor ozone concentration. (**a**) Temperature, (**b**) wind speed, (**c**) wind direction, (**d**) ozone deposition velocity.

The effects of different factors on indoor ozone concentration under natural ventilation were also studied, including temperature, wind speed, wind direction, and ozone deposition velocity. Table 4 shows the settings of influencing factors. In this paper, the variable-controlling method was used to study the effects of different influencing factors on indoor ozone concentration. The first row shows the average value of different factors in summer and winter. Using temperature as an example, 25% and 75% percentiles were used to study the effect of temperature on indoor ozone concentration in the living room, the kitchen, and Bedroom A, while other factors remained unchanged. The same was true of other influencing factors. The results are shown in Figure 4.

It can be seen that when the inner doors were closed in summer, decreasing the outdoor temperature from 28 °C to 26 °C reduced the ozone concentration of Bedroom A by 28.3%. In winter, the ozone concentration in the living room was more sensitive to temperature changes. The ozone concentration in the kitchen showed minimal effect by temperature, and it showed a great effect from wind speed, wind direction, and deposition. When inner doors were closed in summer, increasing wind speed, decreasing wind angle, and deposition velocity could lead to a great ozone variation in the kitchen. As for ozone deposition velocity, there was more room for indoor ozone concentration to decrease. Generally speaking, indoor ozone concentration in winter was affected more by the above factors than in summer.

### 3.3. Indoor Ozone Level with Ozone Emission Devices.

This section mainly studied the ozone concentration level of each room when there were ozone emission devices in the building. According to the questionnaire survey, we used CONTAM to simulate the indoor ozone concentration hourly when photocopying devices were located in Bedroom A and the living room, when air purifying devices were in Bedroom A and the living room, and when disinfecting devices were in the kitchen. The photocopying devices worked from 11:00 to 14:00 on Monday and Wednesday and from 14:00 to 17:00 on Friday and Sunday. The median ozone emission rate 5 mg/h was adopted. The air purifying devices worked from 20:00 to 24:00 every day, and the median ozone emission rate 38.8 mg/h was adopted. The disinfecting devices worked from 11:00 to 14:00 and from 17:00 to 20:00 on Friday and Saturday, and the median ozone emission rate 14.4 mg/h was adopted. The simulation time step was 5 min, and the total duration was 3 months in summer or winter. Whether the inner doors were closed or open was also considered in this section.

**Figure 5:**
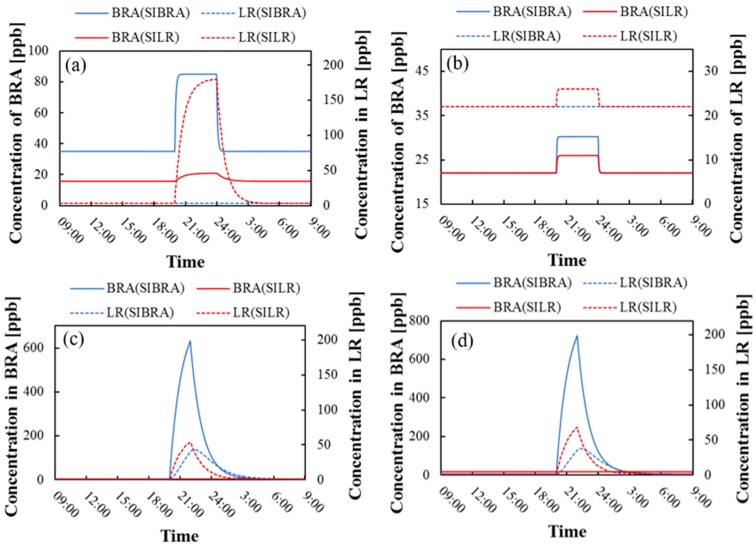
Ozone sources in Bedroom A and the living room on the condition that (**a**) inner doors were closed in summer, (**b**) inner doors were open in summer, (**c**) inner doors were closed in winter, (**d**) inner doors were open in winter. BRA and LR refer to the ozone concentration of Bedroom A and the living room, respectively. SIBRA and SILR mean that sources were located in Bedroom A and the living room respectively.

Taking the air purifying devices as an example, this paper studied the variation of the ozone concentration level of the source room and the adjacent representative room when ozone was emitted, as shown in Figure 5.

Figure 5 shows the hourly variation of ozone concentration in Bedroom A and the living room on a day when indoor sources were located in one of them. When the ozone emission devices started working at 20:00 h, the concentration of the source room would have a significant and sudden increase, while the other room would change slightly or would remain unchanged. This was because ozone could spread to other rooms through inner doors or cracks, but when the source room was located in the downwind direction, it had less impact on other rooms, just like the case where the sources were located in the living room [LR(SIBRA)] in Figure 5a,b. Taking Figure 5a as an example, when inner doors were closed in summer and the source was located in Bedroom A, it took about 30 min for Bedroom A to reach the maximum 85 ppb, which was consistent with the results of literature [32]. After the devices were shut down, its concentration returned to the background concentration rapidly. The ozone concentration in Bedroom A exceeded 50 ppb for three hours, thus threatening the health of residents. In winter, the ozone concentration in source rooms was particularly high, indicating a need for additional attention.

Figure 5 shows the ozone level for only one day when air purifying devices were located in the building. Different types of devices and different source locations were also simulated hourly. The average value is shown in Table 5. It can be seen that when inner doors were open in summer, the ozone level was nearly two times higher than that with inner doors closed, except in Bedroom A. In winter, the state of inner doors had little effect.

When there were ozone emission devices in the building, the indoor ozone was from the combination of indoor and outdoor sources. The conditions with indoor ozone sources would also be the basis for the calculation of the contributions of the sources.

### 3.4. Indoor and Outdoor Ozone Contributions

In this paper, three methods were used to quantify and compare the contributions of indoor and outdoor ozone sources. The most commonly used method is to calculate the average I/O ratio of the building. According to the simulation in previous sections, average indoor and outdoor concentrations of the building could be obtained. The average I/O ratios of the main rooms are shown in Table 6.

When there were no ozone sources in the building, the I/O ratio of the living room, Bedroom A, and the kitchen reached more than 0.7 when inner doors were open in summer. However, in winter, no matter whether the inner doors were closed or open, the I/O ratio of each room was small. The I/O ratio of Bedroom A decreased when inner doors were open, whereas other rooms increased. This meant that Bedroom A was most disadvantageous to ozone spread. When there were ozone emission devices in the building, the ozone level of all scenarios had a certain increase, especially in winter. The ozone level of the source room was greater than that of the building average, and this was due to the fact that the indoor ozone in other rooms was all spread from the source room through the inner doors or the cracks. As for different ozone emission devices, they contributed almost the same to indoor ozone in summer, but in winter, air purifying devices contributed the most of the three kinds of devices. Only when the air purifying devices were located in Bedroom A in winter was the I/O ratio greater than 1, meaning that indoor sources contributed more than outdoor sources. In this case, residents should control the use of ozone emission devices and open windows for reducing ozone exposure. In addition to such a condition, when I/O was less than 1, the contribution of outdoor sources was greater than that of indoor sources.

**Figure 6:**
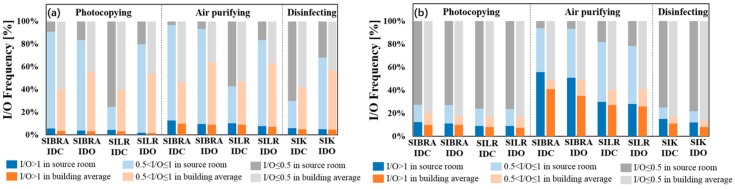
I/O ratio frequency of source room and building average (**a**) in summer, (**b**) in winter.

Average I/O ratio could only roughly show the indoor and the outdoor ozone concentration, since it was affected by many other factors. Therefore, in order to make a more detailed analysis, the ozone I/O ratios of Bedroom A and the living room in summer (1 June–31 August) and winter (1 December–28 February) were calculated hourly, and the frequency of I/O ratio in different ranges is shown in Figure 6.

The darker bar indicates that the I/O ratio was greater than 1, i.e., the concentration indoors was higher than that outdoors. In summer, no matter which kind of devices or in which room the ozone emission devices were located, the frequencies of I/O > 1 of both the source room and the building average were no more than 13%, indicating that outdoor sources were absolutely dominant. Among the three kinds of devices, air purifying devices had the highest frequency of IO > 1. Moreover, the difference between scenarios of the same devices was not significant for I/O > 1. In winter, the frequency of I/O > 1 was higher than that in summer, accounting for 7.5–55.8%, but only when air purifying devices were located in Bedroom A could the frequency of I/O > 1 exceed 50%, meaning that indoor sources became dominant. The result was the same with the method of calculating the average I/O ratio, but the I/O frequency contained more information.

**Figure 7:**
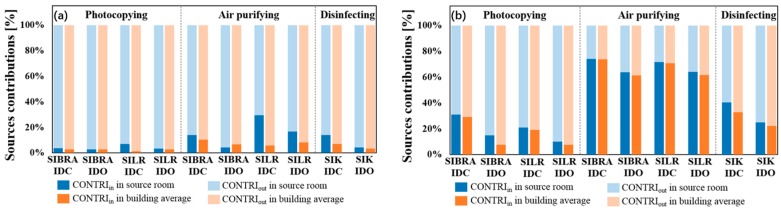
Contributions of indoor and outdoor sources (**a**) in summer, (**b**) in winter. SIBRA, SILR and SIK refer to sources that are located in Bedroom A, the living room, and the kitchen, respectively. CONTRI_in_ and CONTRI_out_ refer to indoor and outdoor sources contributions.

In addition to the analysis of I/O, the ratio of concentrations in the building without and with ozone sources could also be used to quantify and compare the contributions of indoor and outdoor sources. Table 3 and Table 5 calculate the ozone concentrations of the main rooms without and with ozone sources. By dividing the concentration of the corresponding rooms, the contributions of both indoor and outdoor sources could be calculated, as shown in Figure 7.

As can be seen in Figure 7, in summer, no matter which kind of ozone emission devices or in which room the indoor sources were located, the contributions of outdoor sources were absolutely dominant, all exceeding 70%. The highest indoor source contribution also occurred in air purifying devices. When inner doors were closed, the contributions of indoor sources were greater on the same condition. In winter, the contributions of indoor sources had an increase, with most contributions coming from photocopying devices at 30.9%, disinfecting devices at 40.5%, and air purifying devices at more than 50%, showing that indoor sources were dominant.

Comparing the conclusions of Figure 6 and Figure 7, outdoor sources dominated in summer in both methods. In winter, only when air purifying devices were located in Bedroom A could the frequency of I/O > 1 exceed 50%, while the contribution of indoor sources could exceed that of outdoor sources in both Bedroom A and the living room, according to Figure 7. This was because I/O frequencies were calculated by counting the hours’ proportion of different I/O ranges, but there might have been a very large I/O in a short period of time, which may have led to a high average concentration but only a little improvement on I/O > 1 time. Both of these results make sense in quantification and comparison of indoor and outdoor ozone contributions.

### 3.5. Human Respiratory Exposure

The total exposure of the human body to air pollutants refers to the product of pollutant concentration and the residence time when a person stays in a specific environment. Indoor air pollutant exposure could be calculated by the Equation (4) [33,34,35]:

(4) $$\mathrm{BE}=\int_{t_{1}}^{t_{2}}c\left(t\right)dt$$

where BE is total exposure (mg), c is exposure concentration (mg/m^3^), and t_1_ and t_2_ are exposure start and stop time(s), respectively. Total exposure includes respiratory exposure and skin exposure. This paper only considered respiratory exposure. There is a big difference in respiratory rate under different conditions, thus the effect of the respiratory rate on exposure should be considered. Long-term respiratory volume could be calculated by Equation (5) [36,37,38]:

(5) $$IR_{L}=\frac{BMR\times E\times VQ\times A}{1000}$$

where IR_L_ is long-term respiratory volume (m^3^/d), BMR is basal metabolic rate (kJ/d), E is oxygen consumption by unit energy metabolic (giving us 0.05 L/kJ), VQ is the dimensionless ventilation equivalent of 27 here, and A is the coefficient of long-term respiratory volume. BMR and A of different sex and age groups can be found in technical specifications according to height and weight. In this paper, the condition that air purifying devices were located in the building was used to study the ozone respiratory exposure.

**Figure 8:**
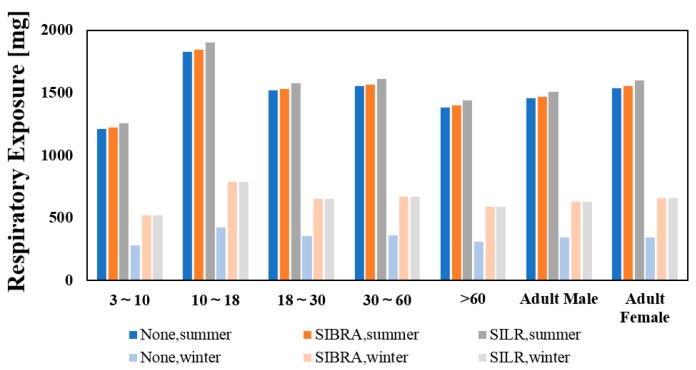
Respiratory exposure in different ages and genders.

Figure 8 shows the respiratory exposure in different ages and genders. It was found that teenagers aged 10–18 years old had the highest respiratory exposure. This was because teenagers had a higher respiratory coefficient, even though they were smaller in weight. Considering that teenagers are more sensitive to air pollutants, relevant control strategies should pay more attention to their protection. The ozone exposure of adult females was higher than that of adult males. Although males had a higher long-term respiratory rate, their indoor activity time was shorter than that of females.

### 3.6. Implications and Limitations of the Study

In this study, three methods were used to quantitatively estimate the contributions of indoor and outdoor sources to indoor ozone in residential buildings. Although indoor ozone levels vary with seasons, with inner doors open or closed, with meteorological parameters, as well as with different settings of ozone emission devices, indoor source contributions to indoor ozone cannot be ignored for most scenarios, especially in winter. Some studies simply utilized ambient ozone concentrations to evaluate human exposure [39,40], which may not reflect the real exposure concentration according to the results of this paper. As to the implications of this study, the results of the model for a typical residence in Nanjing revealed the significance of indoor ozone contributions, thus calling attention to the need for increased scrutiny of indoor sources of ozone. In addition, different control strategies such as closing or opening doors and windows in a timely manner or using ozone emission devices can be taken in different scenarios to minimize the human exposure to ozone and to provide for a healthier building environment.

However, there are some limitations of this research and the model implications at present. For example, the building model used in this paper represents only the most typical situation in Nanjing. Since there are so many types of residential buildings in the city, they all cannot be fully covered in this paper. However, the research methods and the results can be generalized in this region. Additionally, as a pure software simulation research, if there were real experiments for comparison, the results would be more credible and scientific. The next step of this study is to select some typical residential buildings in Nanjing to monitor the ozone concentration over a longer period of time and to record the residents’ customary practices of opening and closing windows and doors so as to provide experimental support for this research. In addition to indoor and outdoor ozone sources, indoor ozone concentration is also affected by ozone removal rate. The indoor ozone concentration can be defined by Equation (6) [15].

(6) $$\frac{{\mathrm{dC}}_{\mathrm{in}}}{\mathrm{dt}}={k\lambda C}_{\mathrm{out}}+R_{E}-{\lambda C}_{\mathrm{in}}-\sum^{\;}R_{C}-\sum^{\;}R_{M}-\sum^{\;}R_{H}$$

where C_in_ and C_out_ are the indoor and the outdoor ozone concentration (ppb), k is the ozone penetration factor (0–1), λ is the air exchange rate (h-1), RE is the ozone emission rate of the indoor ozone devices (ppb/h), RC is the ozone removal rate by gaseous chemicals through chemical reactions (ppb/h), RM is the ozone removal rate by indoor building materials (ppb/h), and RH is the rate at which ozone is removed by human surfaces (ppb/h). The ozone removal rate on the surface of the building material and the human body was kept constant in this paper, but the chemical reaction between ozone and other gases was not considered, because it is too complex and the amount of reaction is small. Future studies should investigate the impact of different kinds of ozone removal in residential buildings in detail.

## 4. Conclusions

This study aimed to investigate the contributions of indoor and outdoor sources to ozone in residential buildings. The multi-zone software CONTAM was used to simulate the air exchange rate and the ozone concentration in a typical residence in Nanjing, China. Firstly, a questionnaire survey about the ozone emission devices was performed in Nanjing. The devices type, the placement, and the frequency of use analyzed by the questionnaire survey provided the basis for the settings of indoor ozone sources. Then, the natural ventilation pattern of the typical building model was simulated, and air exchange rates and ozone concentrations of the main rooms were obtained. The natural ventilation condition with no indoor sources served as the basis of the calculation of source contributions. The effects of different factors on indoor ozone concentration under natural ventilation were also studied, including temperature, wind speed, wind direction, and ozone deposition velocity. Next, the condition under which there were ozone emission devices indoors was simulated. Taking the air purifying devices as an example, this paper studied the variations of ozone concentration levels of the source room and the adjacent representative room when ozone was emitted. Meanwhile, the average ozone concentrations in the source room and the building average with different ozone emission devices were calculated.

Three methods were used to quantify the contribution of indoor and outdoor ozone sources in this paper, namely, the average I/O ratio, the frequency of I/O, and the ratio of indoor ozone concentration without ozone sources to that with ozone sources. According to average I/O ratio, only when air purifying devices were located in Bedroom A in winter could the indoor source contribution exceed the outdoor. I/O frequency was the proportion of the time when I/O was greater than 1, less than 0.5, and between 0.5 and 1 in the whole season. It was found by I/O frequency analysis that outdoor sources were dominant in summer, but when air purifying devices were located in Bedroom A in winter, the frequency of I/O > 1 could exceed 50%, which meant that indoor sources became dominant. The ratio of indoor ozone concentration without ozone sources to that with ozone sources was the contribution of outdoor sources. The results showed that the contributions of outdoor sources were absolutely dominant in summer, but in winter, the indoor contributions of air purifying devices were more than 50% regardless of the scenarios, meaning that the indoor sources were dominant.

Finally, respiratory exposure in different ages and genders was calculated. Teenagers aged 10–18 had the highest respiratory exposure because of the high respiratory coefficient, and the exposure of female adults was higher than males because they spent more time indoors. The results of the model for a typical residence in Nanjing revealed the significance of indoor ozone contributions, thus calling attention to the need for increased scrutiny of indoor sources of ozone. In addition, appropriate measures should be taken to minimize the human exposure to ozone both indoors and outdoors.

## Figures and Tables

**Table 1:** The size and the effective area of each opening.

Opening	Width (m)	Height (m)	Area (m^2^)	Effective Area When Closed (cm^2^)	Effective Area When Opened (m^2^)	Relative Height (m)
W1	1.2	1.5	1.8	56.7	0.9	1.65
W2, W3	0.9	1.5	1.35	42.5	0.675	1.65
W4	0.6	1.45	0.87	27.4	0.87	1.625
W5	1.8	1.5	2.7	85.0	1.35	1.65
W6	1.2	1.5	1.8	56.7	0.9	1.65
W7	3.6	1.5	5.4	170.1	2.7	1.65
D1	1.8	2	3.6	54	1.8	1
D2, D3, D4	0.9	2	1.8	261	1.8	1
D6	3.6	2	7.2	108	7.2	1
D7	1	2.2	2.2	28.87	2.2	1.1

D and W refer to doors and windows, and their serial number corresponds to Figure 1.

**Table 2:** Questionnaire survey on indoor ozone emission devices.

No.	Question	Options
1	Do you have ozone emission devices in your home?	a. Yes; b. No; c. Uncertain;
2	What type of the devices?	a. Photocopying; b. Air purifying; c. Disinfecting; e. Others; Model Number: ________________
3	Where do you place the ozone emission devices?	a. Living room; b. Bedroom; c. Kitchen; d. Bathroom; e. Balcony; f. Others;
4	How often do you use the ozone emission devices?	a. Every day; b. 1–2 times a week; c. 3–4 times a week; d. 5–6 times a week; e. Uncertain;
5	When do you use the ozone emission devices?	a. 5:00–9:00; b. 9:00–11:00; c. 11:00–14:00; d. 14:00–16:00; e. 16:00–22:00; f. 22:00–5:00; g. Uncertain;
6	Do you understand that electrical appliances may produce ozone?	a. Good understanding; b. General understanding; c. No understanding.

**Table 3:** Average air exchange rate and ozone concentration in the main rooms when there were no indoor sources.

Room Parameters	Room	Summer		Winter	
		IDC	IDO	IDC	IDO
Air exchange rate (h^−1^)	Living Room	0.68	50.93	0.21	0.26
	Bedroom A	21.91	78.58	0.28	0.33
	Kitchen	0.23	43.48	0.17	0.25
	Building average	3.03	20.74	0.09	0.11
Ozone concentration (ppb)	Living Room	13	34	1.3	1.4
	Bedroom A	43	39	2.6	2.8
	Kitchen	8	36	1.8	2.3
	Building Average	14	30	1.2	1.4

IDC and IDO refer to inner doors closed and inner doors open, respectively.

**Table 4:** Influencing factors settings.

Influencing Factors	Season	Temperature (°C)	Wind Speed (m/s)	Wind Direction (°)	Ozone Deposition Velocity (cm/s)
Average values of the factors	Summer	28	2.45	135	0.054
	Winter	5	2.45	45	0.054
Temperature	Summer	26	2.45	135	0.054
		30			
	Winter	2	2.45	45	
		8			
Wind speed	Summer	28	0.9	135	0.054
			4.4		
	Winter	5	0.9	45	
			4.4		
Wind direction	Summer	28	2.45	112.5	0.054
				157.5	
	Winter	5		22.5	
				67.5	
Ozone deposition velocity	Summer	28	2.45	135	0.02
					0.08
	Winter	5		45	0.02
					0.08

**Table 5:** Average ozone concentration in the source room and the building average when there were ozone emission devices indoors.

Scenarios	Room	Summer (ppb)		Winter (ppb)	
		IDC	IDO	IDC	IDO
Photocopying devices in BRA	Bedroom A	42.7	38.1	3.8	3.9
	Building average	14.4	29.8	1.4	1.5
Photocopying devices in LR	Living Room	13	34.4	1.6	1.7
	Building average	14.5	29.8	1.3	1.5
Air purifying devices in BRA	Bedroom A	43.9	39.5	10.1	10.7
	Building average	14.6	30.1	3.3	3.6
Air purifying devices in LR	Living Room	18.4	36.1	4.6	4.8
	Building average	16.8	30.7	3.3	3.6
Disinfecting devices in Kitchen	Kitchen	9.3	35.7	3.0	3.4
	Building average	14.6	29.8	1.6	1.8

**Table 6:** Average indoor/outdoor (I/O) ratio of the main rooms.

Scenarios	Room	Summer (ppb)		Winter (ppb)	
		IDC	IDO	IDC	IDO
No indoor sources	Living Room	0.26	0.71	0.07	0.07
	Bedroom A	0.88	0.79	0.13	0.14
	Kitchen	0.16	0.74	0.09	0.12
	Building Average	0.3	0.62	0.06	0.07
Photocopying devices in BRA	Bedroom A	0.89	0.80	0.27	0.28
	Building average	0.3	0.62	0.08	0.09
Photocopying devices in LR	Living Room	0.27	0.72	0.11	0.12
	Building average	0.3	0.62	0.08	0.09
Air purifying devices in BRA	Bedroom A	0.91	0.82	2.34	2.33
	Building average	0.3	0.62	0.38	0.39
Air purifying devices in LR	Living Room	0.38	0.75	0.8	0.79
	Building average	0.35	0.63	0.39	0.39
Disinfecting devices in Kitchen	Kitchen	0.19	0.75	0.17	0.19
	Building average	0.3	0.62	0.08	0.09
